# Supplementary material for: Potential Effects of 25-Hydroxycholecalciferol on the Growth Performance, Blood Antioxidant Capacity, Intestinal Barrier Function and Microbiota in Broilers under Lipopolysaccharide Challenge
Source: Antioxidants (Basel). 2022 Oct 24;11(11):2094. doi: 10.3390/antiox11112094 (PMC9686511; doi:10.3390/antiox11112094)
Supplement: Supplementary file 1 [file antioxidants-11-02094-s001.zip › antioxidants-1921501-supplementary.pdf]

Supplementary Materials

Table S1. Effects of 25OHD3 supplementation on growth performance of broilers before LPS challenge.[1.]

Item	CON	LPS	LPS + 25OHD3	SEM	*P*-value
Initial body weight, g	48.69	48.55	48.19	0.81	0.90
Final body weight, g	690.36	698.75	673.80	19.07	0.65
Average daily gain, g	30.56	30.96	29.79	1.00	0.71
Average daily feed intake, g	43.19	43.32	41.30	2.12	0.76
Feed:gain	1.41	1.40	1.38	0.04	0.92

CON, control; LPS, lipopolysaccharide; LPS + 25OHD3, lipopolysaccharide + 25-hydroxycholecalciferol.[1] Data are present as means and SEM (n = 6).
